# Dietary inulin affects the intestinal microbiota in sows and their suckling piglets

**DOI:** 10.1186/s12917-015-0351-7

**Published:** 2015-03-07

**Authors:** Nadine Paßlack, Wilfried Vahjen, Jürgen Zentek

**Affiliations:** Department of Veterinary Medicine, Institute of Animal Nutrition, Freie Universität Berlin, Königin-Luise-Str. 49, 14195 Berlin, Germany

**Keywords:** Inulin, Intestinal microbiota, Sows, Suckling piglets, Enterococci

## Abstract

**Background:**

Several studies have focused on the effects of dietary inulin on the intestinal microbiota of weaned piglets. In the present study, inulin was added to a diet for gestating and lactating sows, expecting not only effects on the faecal microbiota of sows, but also on the bacterial cell numbers in the gastrointestinal tract of their piglets during the suckling period. Sows were fed a diet without (n = 11) or with (n = 10) 3% inulin, and selected bacterial groups were determined in their faeces ante and post partum. Suckling piglets, 8 per group, were euthanised on day 10 after birth to analyse digesta samples of the gastrointestinal tract.

**Results:**

Dietary inulin increased the cell numbers of enterococci, both, in the faeces of the sows during gestation and lactation, and in the caecum of the piglets (*P* ≤ 0.05). Moreover, higher cell numbers of eubacteria (stomach) and *C. leptum* (caecum), but lower cell numbers of enterobacteria and *L. amylovorus* (stomach) were detected in the digesta of the piglets in the inulin group (*P* ≤ 0.05).

**Conclusions:**

In conclusion, inulin seems to have the potential to influence the gastrointestinal microbiota of suckling piglets through the diet of their mother, showing the importance of the mother-piglet couple for the microbial development. Early modulation of the intestinal microbiota could be especially interesting with regard to the critical weaning time.

## Background

The polyfructan inulin is considered to act as a prebiotic, since it can modulate the composition and metabolic activity of the intestinal microbiota, which might potentially enhance the health of the host organism [1-3]. Several studies have demonstrated that inulin can modulate the intestinal microbiota in pigs. In particular, bifidobacteria and lactobacilli were increased in different segments of the intestinal tract, when inulin was added in a concentration of 1.6% [4] and 4% [5,6] to the diets. Moreover, a decrease of *Clostridium perfringens* in the porcine digesta of the colon and rectum [7] or *Clostridium* spp. and members of *Enterobacteriaceae* in the porcine digesta and mucus in different segments of the intestine [6] was reported after the dietary inclusion of inulin. These results are conflicting, as other studies could not demonstrate an effect of dietary inulin on the intestinal microbiota in piglets [8,9].

To our best knowledge, no studies have been conducted to evaluate the effects of dietary inulin in gestating and lactating sows. However, this aspect might be interesting, since a modulation of the intestinal microbiota of the mother sows might also affect the bacterial community in the intestinal tract of their piglets. This sow-piglet-axis is particularly relevant, since the physiological condition of suckling piglets is closely connected with the mother sows in the first weeks of life, including energy and nutrient supply [10-12], immunological protection [13] and the microbial colonisation of the gastrointestinal tract [14]. Previous studies have demonstrated that a probiotic treatment of sows altered the composition of the intestinal microbiota in their offspring [15-17]. However, no study has evaluated the potential of prebiotics with regard to the sow-piglet-axis up to now.

It was the aim of the present study to determine the effects of dietary inulin on the composition and metabolic activity of the intestinal microbiota of gestating and lactating sows and their suckling piglets.

## Results

### Animal health and reproductive parameters

All animals were healthy throughout the sampling period. No significant group differences were observed for the measured reproductive parameters, however, sows of the inulin group had a numerically higher number of piglets per litter compared to the control group (*P* > 0.05). The total number of piglets per litter was 10.2 in the control group and 11.3 in the inulin group (*P* > 0.05). The number of piglets which were born alive and stillborn was 9.18 and 1.00 in the control group and 10.8 and 0.50 in the inulin group (*P* > 0.05). In addition, numerically more piglets were weaned in the inulin group compared to the control group, with 9.60 and 8.00 piglets per litter (*P* > 0.05).

### Bacterial cell counts in the faeces of the sows

Except for the cell numbers of enterococci, no bacterial group in the faeces of the sows was systematically influenced by the dietary inclusion of inulin (Table 1). Enterococci were higher in the inulin group compared to the control group at all measuring times (*P* = 0.014). A time effect was observed for the cell numbers of eubacteria, lactobacilli, *L. reuteri*, *L. amylovorus*, *L. johnsonii*, *L. mucosae*, *C. leptum*, and *C. coccoides*, which were the lowest on day 1 *post partum* (p.p.) compared to the days 4 *ante partum* (a.p.) and 5 p.p. (*P ≤* 0.05).

**Table 1:** **Microbial cell counts (log**_**10**_ **/g wet weight) in the faeces of sows fed a diet without (C) or with inulin (I)**

	**Day 4 a.p.**		**Day 1 p.p.**		**Day 5 p.p.**		***P*** **-value**		
	**C (n** ^**1**^ **= 10)**	**I (n = 10)**	**C (n = 10)**	**I (n = 9)**	**C (n = 10)**	**I (n = 10)**	**Diet**	**Time**	Diet*time
Eubacteria	10.6 ± 0.51	10.8 ± 0.85	9.86 ± 0.10	9.75 ± 0.21	10.8 ± 0.15	10.8 ± 0.18	0.847	**0.005**	0.718
Enterobacteria	7.21 ± 1.01	6.66 ± 0.90	7.16 ± 0.98	7.77 ± 0.75	7.42 ± 0.68	7.54 ± 0.99	0.802	0.103	**0.017**
Enterococci	6.42 ± 0.45	6.83 ± 0.59	5.96 ± 0.31	7.00 ± 0.91	6.62 ± 0.48	6.98 ± 0.61	**0.014**	0.109	**0.028**
Bifidobacteria	7.92 ± 1.05	8.10 ± 1.91	7.24 ± 0.51	7.60 ± 1.08	8.05 ± 0.87	7.92 ± 1.51	0.976	0.052	0.357
Lactobacilli	9.39 ± 0.62	9.32 ± 0.77	8.89 ± 0.67	7.73 ± 1.42	8.98 ± 0.57	8.60 ± 0.89	0.109	**<0.001**	0.051
*L. reuteri*	8.37 ± 0.86	8.25 ± 0.64	7.36 ± 1.12	6.83 ± 0.66	7.93 ± 0.73	7.57 ± 0.47	0.305	**<0.001**	0.244
*L. amylovorus*	9.39 ± 0.40	9.32 ± 0.35	8.50 ± 0.96	8.22 ± 0.63	9.12 ± 0.61	9.03 ± 0.32	0.502	**<0.001**	0.606
*L. johnsonii*	6.16 ± 1.10	5.81 ± 0.94	4.97 ± 0.79	5.20 ± 0.52	5.46 ± 0.42	5.25 ± 0.48	0.872	**0.002**	0.411
*L. mucosae*	8.22 ± 0.83	7.83 ± 0.41	7.08 ± 0.89	6.72 ± 0.47	7.56 ± 0.61	7.17 ± 0.96	0.088	**<0.001**	0.898
*C. leptum*	10.5 ± 0.24	10.1 ± 0.55	9.50 ± 0.97	9.84 ± 0.62	9.89 ± 0.73	9.91 ± 1.11	0.987	**0.010**	0.266
*C. coccoides*	10.7 ± 0.53	10.8 ± 0.24	9.69 ± 1.05	9.79 ± 0.77	10.6 ± 0.41	10.5 ± 0.93	0.891	**0.002**	0.762
BPP	9.66 ± 0.53	9.69 ± 0.54	9.40 ± 0.25	9.31 ± 0.37	9.83 ± 0.52	9.55 ± 0.28	0.152	0.052	0.557

^1^Available sample size for analysis: For Eubacteria: day 4 *a.p.*: n = 5 (C) and n = 5 (I); day 1 *p.p.*: n = 5 (C) and n = 4 (I); day 5 *p.p.*: n = 5 (C) and n = 5 (I); for Enterobacteria: day 4 *a.p.*: n = 8 (C) and n = 10 (I); day 1 *p.p.*: n = 8 (C) and n = 9 (I); day 5 *p.p.*: n = 9 (C) and n = 10 (I); for *L. amylovorus*: day 4 *a.p.*: n = 10 (C) and n = 9 (I); day 1 *p.p.*: n = 10 (C) and n = 9 (I); day 5 *p.p.*: n = 9 (C) and n = 10 (I); for *L. johnsonii*: day 4 *a.p.*: n = 10 (C) and n = 10 (I); day 1 *p.p.*: n = 10 (C) and n = 9 (I); day 5 *p.p.*: n = 8 (C) and n = 10 (I).

*Abbreviations:* a.p.: *ante partum*; *BPP* Bacteroides-Prevotella-Porphyromonas Cluster; p.p.: *post partum*.

Boldface *P*-values indicate significant effects (*P* ≤ 0.05).

### pH and bacterial metabolites in the faeces of the sows

The faecal pH was lower (*P* = 0.007) in the inulin group compared to the control group at all measuring times (Table 2). Time-dependent changes were observed for the bacterial metabolites in the faeces of the sows, with higher concentrations of D- and L-lactate (both *P =* 0.002) and propionic acid (*P* < 0.001) on day 4 a.p. compared to the days 1 and 5 p.p. In contrast, the amounts of acetic acid were lower (*P* < 0.001) on day 4 a.p. compared to the days 1 and 5 p.p. The total concentrations of short chain fatty acids (SCFA) and the concentrations of ammonia were high on day 4 a.p., decreased on day 1 p.p. and subsequently increased until day 5 p.p. (*P <* 0.001 and *P* = 0.001 for SCFA and ammonia, respectively). The concentrations of i-butyric acid (*P* = 0.037), n-butyric acid (*P* = 0.025) and n-valeric acid (*P* = 0.012) were the lowest on day 5 p.p. when compared to the other measuring times.

**Table 2:** **Microbial metabolites and pH in the faeces of sows fed a diet without (C) or with inulin (I)**

	**Day 4 a.p.**		**Day 1 p.p.**		**Day 5 p.p.**		***P*** **-value**		
	**C (n = 11)**	**I (n = 10)**	**C (n = 11)**	**I (n = 9)**	**C (n = 11)**	**I (n = 10)**	**Diet**	**Time**	Diet*time
pH	6.79 ± 0.23	6.63 ± 0.34	6.99 ± 0.34	6.59 ± 0.36	6.68 ± 0.12	6.63 ± 0.23	**0.007**	0.323	0.167
L-lactate (mmol/kg)	0.73 ± 0.55	0.70 ± 0.30	0.43 ± 0.28	0.28 ± 0.22	0.35 ± 0.18	0.24 ± 0.19	0.185	**0.002**	0.909
D-lactate (mmol/kg)	0.62 ± 0.64	0.54 ± 0.26	0.20 ± 0.20	0.14 ± 0.12	0.16 ± 0.12	0.11 ± 0.11	0.451	**0.002**	0.947
Ammonia (mmol/kg)	32.6 ± 19.5	47.0 ± 29.1	13.7 ± 9.64	20.2 ± 10.4	21.7 ± 10.2	27.3 ± 12.9	0.070	**0.001**	0.586
SCFA (mmol/l)	152 ± 44.4	155 ± 30.1	100 ± 31.4	123 ± 31.5	138 ± 38.3	158 ± 17.5	0.164	**<0.001**	0.602
Acetic acid (mol. %)	54.4 ± 3.61	53.7 ± 3.78	60.6 ± 4.11	56.8 ± 4.19	60.9 ± 4.35	59.1 ± 3.16	0.081	**<0.001**	0.388
Propionic acid (mol. %)	23.0 ± 1.08	22.6 ± 1.33	19.3 ± 2.03	20.9 ± 1.11	19.4 ± 1.40	19.9 ± 1.69	0.153	**<0.001**	0.053
i-butyric acid (mol. %)	2.87 ± 0.56	2.79 ± 0.33	2.87 ± 0.40	2.61 ± 0.55	2.52 ± 0.27	2.51 ± 0.32	0.337	**0.037**	0.641
n-butyric acid (mol.%)	13.0 ± 2.85	13.9 ± 2.62	10.4 ± 4.24	12.8 ± 3.36	11.1 ± 3.54	12.4 ± 1.71	0.152	**0.025**	0.711
i-valeric acid (mol. %)	4.05 ± 0.91	3.92 ± 0.51	4.06 ± 0.62	3.88 ± 0.86	3.62 ± 0.43	3.56 ± 0.53	0.538	0.068	0.950
n-valeric acid (mol. %)	2.66 ± 0.50	3.09 ± 0.70	2.77 ± 0.51	3.02 ± 0.63	2.45 ± 0.51	2.61 ± 0.45	0.147	**0.012**	0.467

*Abbreviations:* a.p.: *ante partum*; p.p.: *post partum*; *SCFA* short chain fatty acids.

Boldface *P*-values indicate significant effects (*P* ≤ 0.05).

### Bacterial cell counts in the digesta of the suckling piglets

In the digesta of the stomach of the suckling piglets, cell numbers of eubacteria were higher and cell numbers of enterobacteria and *L. amylovorus* were lower in the inulin group compared to the control group (*P* ≤ 0.05) (Table 3). The cell numbers of enterococci and *C. leptum* were higher in the digesta of the caecum of the inulin group when compared to the control group (*P* ≤ 0.05).

**Table 3:** **Microbial cell counts (log**_**10**_ **/g wet weight) in the digesta of the stomach and caecum of suckling piglets, where the mother sows received either a diet without (C) or with inulin (I)**

	**Stomach**		**Caecum**	
	**C (n** ^**1**^ **= 7)**	**I (n = 8)**	**C (n** ^**2**^ **= 8)**	**I (n = 8)**
Eubacteria	10.3 ± 0.26^a^	11.0 ± 0.25^b^	11.7 ± 0.37	11.7 ± 0.33
Enterobacteria	4.56 ± 0.87^a^	3.45 ± 0.50^b^	9.02 ± 0.66	8.58 ± 1.20
Enterococci	7.24 ± 0.45	7.21 ± 0.74	6.76 ± 0.49^a^	7.57 ± 0.46^b^
Bifidobacteria	3.89 ± 0.70	5.04 ± 1.83	5.16 ± 0.91	5.57 ± 1.29
Lactobacilli	9.31 ± 0.88	9.06 ± 0.97	10.0 ± 1.23	9.94 ± 1.49
*L. reuteri*	8.23 ± 0.63	7.81 ± 0.84	8.40 ± 0.50	8.13 ± 0.62
*L. amylovorus*	9.28 ± 0.32^a^	8.17 ± 0.66^b^	9.26 ± 0.69	9.28 ± 0.79
*L. johnsonii*	7.05 ± 0.69	6.45 ± 0.93	6.76 ± 0.79	6.22 ± 1.09
*L. mucosae*	7.61 ± 0.82	6.78 ± 1.42	7.95 ± 0.89	7.97 ± 1.31
*C. leptum*	6.70 ± 0.31	6.40 ± 0.89	10.0 ± 0.78^a^	10.8 ± 0.15^b^
*C. coccoides*	7.02 ± 0.93	6.66 ± 1.02	10.3 ± 0.78	10.7 ± 0.43
BPP	6.14 ± 1.12	5.43 ± 1.72	9.87 ± 0.74	10.3 ± 0.32

^1^Available sample size for analysis (stomach): For Eubacteria: n = 4 (C) and n = 4 (I); for Lactobacilli and *L. amylovorus*: n = 7 (I).

^2^Available sample size for analysis (caecum): For Eubacteria: n = 4 (C) and n = 4 (I).

Abbreviations: a.p.: *ante partum*; BPP: Bacteroides-Prevotella-Porphyromonas Cluster p.p.: *post partum.*

Different letters in the same row indicate significant differences (*P ≤* 0.05). Group comparisons were calculated separately for stomach and caecum.

### pH and bacterial metabolites in the digesta of the suckling piglets

No group differences were observed for the pH of the digesta in the stomach, small intestine, caecum and rectum of the suckling piglets (*P* > 0.05) (Table 4). The concentrations of ammonia, n-butyric acid and i-valeric acid in the digesta of the stomach were lower in the inulin group when compared to the control group (*P* ≤ 0.05). No group differences were detected for the bacterial metabolites in the digesta of the small intestine, caecum and rectum of the suckling piglets (*P* > 0.05).

**Table 4:** **Microbial metabolites and pH in the digesta of suckling piglets, where the mother sows received a diet without (C) or with inulin (I)**

	**Stomach**		**Small intestine**		**Caecum**		**Rectum**	
	**C (n** ^**1**^ **= 8)**	**I (n = 8)**	**C (n** ^**2**^ **= 8)**	**I (n = 8)**	**C (n** ^**3**^ **= 8)**	**I (n = 8)**	**C (n** ^**4**^ **= 8)**	**I (n = 8)**
pH	3.27 ± 0.44	2.74 ± 0.75	6.78 ± 0.12	6.71 ± 0.64	6.07 ± 0.15	6.20 ± 0.24	6.15 ± 0.49	6.36 ± 0.62
L-lactate (mmol/kg)	12.9 ± 2.68	7.68 ± 8.85	8.25 ± 4.13	7.79 ± 3.86	0.34 ± 0.25	0.25 ± 0.19	0.54 ± 0.83	0.22 ± 0.15
D-lactate (mmol/kg)	16.7 ± 5.64	13.0 ± 16.9	1.40 ± 1.08	1.21 ± 1.21	0.32 ± 0.23	0.22 ± 0.22	0.08 ± 0.07	0.09 ± 0.06
Ammonia (mmol/kg)	3.00 ± 0.92^a^	2.09 ± 0.71^b^	3.95 ± 2.09	3.63 ± 1.94	31.9 ± 13.5	23.4 ± 6.60	13.6 ± 4.70	**
SCFA (mmol/l)	3.90 ± 2.22	3.74 ± 2.14	4.82 ± 2.23	3.57 ± 3.64	55.6 ± 15.0	49.2 ± 17.0	22.8 ± 10.8	15.3 ± 8.07
Acetic acid (mol.%)	3.33 ± 1.75	3.52 ± 1.93	4.40 ± 2.08	3.28 ± 3.41	36.5 ± 9.78	33.9 ± 11.4	13.9 ± 6.75	9.68 ± 4.01
Propionic acid (mol.%)	0.12 ± 0.10	0.12 ± 0.20	0.31 ± 0.35	0.19 ± 0.30	10.8 ± 2.59	8.93 ± 3.26	3.83 ± 2.33	2.80 ± 2.36
i-butyric acid (mol.%)	0.01 ± 0.00^a^	0.01 ± 0.02^b^	0.02 ± 0.02	*	1.24 ± 0.49	0.90 ± 0.40	0.69 ± 0.48	0.47 ± 0.36
n-butyric acid (mol.%)	0.18 ± 0.04^a^	0.06 ± 0.07^b^	0.12 ± 0.11	0.11 ± 0.11	3.74 ± 1.68	3.10 ± 1.50	2.07 ± 1.61	1.21 ± 1.12
i-valeric acid (mol.%)	0.27 ± 0.52^a^	0.03 ± 0.03^b^	0.04 ± 0.03	0.03 ± 0.03	1.62 ± 0.56	1.13 ± 0.41	1.37 ± 0.87	0.86 ± 0.67
n-valeric acid (mol.%)	0.02 ± 0.01	0.00 ± 0.00	0.01 ± 0.01	0.01 ± 0.00	1.70 ± 0.72	1.16 ± 0.50	0.91 ± 0.57	0.47 ± 0.24

^1^Available sample size for analysis (stomach): For pH: n = 4/group; for L-lactate, D-lactate, acetic acid, propionic acid, i-butyric acid, and SCFA: n = 7 (C); for i-valeric acid: n = 7 (C) and n = 7 (I); for n-butyric acid: n = 6 (C); for n-valeric acid: n = 4 (C) and n = 6 (I).

^2^Available sample size for analysis (small intestine): For pH: n = 4/group; for D-lactate: n = 7 (I); for propionic acid: n = 7 (C) and n = 6 (I); *for i-butyric acid: n = 3 (C) and n = 1 (I); for n-butyric acid: n = 6 (C) and n = 7 (I); for n-valeric acid: n = 4 (C) and n = 5 (I); for ammonia: n = 5 (C) and n = 6 (I).

^3^Available sample size for analysis (caecum): For pH: n = 4/group; for D-lactate: n = 7/group; for ammonia: n = 7 (C) and n = 6 (I).

^4^Available sample size for analysis (rectum): For pH: n = 4 (C) and n = 3 (I); for L-lactate: n = 5/group; for D-lactate: n = 6 (C) and n = 5 (I); **for ammonia: n = 5 (C) and n = 2 (I); for acetic acid, propionic acid, n-butyric acid, i-valeric acid and SCFA: n = 7 (C) and n = 6 (I); for i-butyric acid and n-valeric acid: n = 7 (C) and n = 5 (I).

Abbreviation: SCFA: short chain fatty acids.

Different letters in the same row indicate significant differences (*P ≤* 0.05). Group comparisons were calculated separately for stomach, small intestine, caecum and rectum.

## Discussion

The physiological condition of suckling piglets is substantially influenced by their mother sows. Besides the intake of colostrum and milk for delivering energy, nutrients, and, importantly, passive immunity, the close contact between sows and suckling piglets is of relevance for the microbial colonisation of the gastrointestinal tract of the newborns. In particular, the contact with mother’s faeces contributes to this microbial colonisation in their offspring [14], and it can be hypothesised that a nutritional modulation of the intestinal microbiota of mother sows also affects the bacterial community in the gastrointestinal tract of their suckling piglets. This link between the intestinal microbiota of sows and piglets has already been demonstrated after a probiotic treatment of mother sows [15-17], however, to our best knowledge, the potential of prebiotics has not been evaluated in this context up to now.

The present results demonstrated that dietary inulin increased the cell numbers of enterococci in sows’ faeces during the gestation and lactation period (*P* = 0.014). Moreover, a higher cell number of enterococci was also measured in the caecal digesta of the suckling piglets of the inulin treated sows (*P* ≤ 0.05), stressing the connection between the composition of the intestinal microbiota of mothers and their offspring. It should not go unmentioned that previous studies demonstrated decreased numbers of enterococci in the faeces of growing pigs [18] respectively in the colonic digesta of newly weaned piglets [19] when inulin was added to the diets. However, due to the higher cell numbers of enterococci at all measuring time points of the present study, a systematically enhancing effect of dietary inulin on the numbers of enterococci in sows can be assumed.

Except for the numbers of enterococci, the bacterial groups in the faeces of the sows were not affected by the dietary inclusion of inulin (*P* > 0.05). In particular, no differences in the cell numbers of bifidobacteria and lactobacilli were observed between the control and inulin group, which is in contrast to previous studies [4-6]. Bifidobacteria and lactobacilli are typically anticipated to be increased by the dietary inclusion of inulin [20]. However, the missing effect in the present study underlines other conflicting results reported in the literature, as some authors also could not demonstrate an effect of dietary inulin on bifidobacteria and lactobacilli [18,19] or generally on the intestinal microbiota in pigs [8,9].

Interestingly, not only the cell numbers of enterococci, but also of some other bacterial groups in the digesta of the stomach and caecum of the suckling piglets differed depending on the dietary treatment of the mother sows. Higher cell numbers of eubacteria (stomach) and *C. leptum* (caecum), but lower cell numbers of enterobacteria and *L. amylovorus* (stomach) were detected in the inulin group when compared to the control group (*P* ≤ 0.05). Up to now, the reason for this observation remains unclear. As environmental conditions were kept similar between the inulin and control group and no additional feed was offered to the suckling piglets, exogenous factors might be excluded. In general, it should be considered that the study design did not allow an evaluation of digesta samples of the sows, particularly samples of the stomach or caecum, why comparisons between the gastrointestinal microbiota of the sows and piglets are limited. It might be that inulin also affected further bacterial groups in the gastrointestinal tract of the sows, but that the analysis of the faeces cannot completely reflect this dietary impact on the microbiota.

Another explanation for the differences between the faecal microbiota of the sows and the gastrointestinal microbiota of the suckling piglets might be individual differences in the intestinal bacterial cell counts of the sows prior to the dietary inulin treatment. This factor was also assumed in the study of Starke et al. [17], where a probiotic *Enterococcus faecium* strain not only affected the bacterial cell numbers in the faeces of mother sows. The authors [17] also observed differences in the intestinal microbiota of suckling piglets, when the offspring of the probiotic and non-probiotic treated sows was compared. However, the probiotic *Enterococcus faecium* strain did not modify the intestinal microbiota of sows and piglets in an equal manner.

Only small effects of dietary inulin on the metabolic activity of the intestinal microbiota of sows and piglets were observed. However, the composition of the microbiota and the concentrations of the microbial metabolites in the faeces of the sows markedly differed depending on the reproductive stage. The bacterial cell counts were often reduced on day 1 p.p. compared to the days 4 a.p. and 5 p.p. Significant differences (*P ≤* 0.05) were demonstrated for eubacteria, lactobacilli, *L. reuteri*, *L. amylovorus*, *L. johnsonii*, *L. mucosae*, *C. leptum*, and *C. coccoides* in both groups*.* The reduced numbers of bacteria could be due to a lower feed intake before and after farrowing, which is commonly found in practice and was also observed in the present study. Time-dependent changes were also detected for the concentrations of the bacterial metabolites in the faeces of the sows. Higher concentrations of lactate (*P* = 0.002) and propionic acid (*P* < 0.001) were measured on day 4 a.p. compared to the days 1 and 5 p.p., and lower concentrations of acetic acid (*P* < 0.001) were detected on day 4 a.p. compared to the days 1 and 5 p.p. The total concentrations of SCFA (*P* <0.001) and the amounts of ammonia (*P* = 0.001) were high on day 4 a.p., decreased until day 1 p.p. and subsequently increased until day 5 p.p. Overall, it can be concluded that the faecal microbiota of the sows might be affected by changes in the feeding regimen during the parturition time, and adapted to increasing intakes of the lactation diet during the first days after farrowing.

## Conclusions

The present results indicate that the addition of inulin to a gestation and lactation diet can not only modulate the intestinal microbiota of sows, but also of their offspring. A promotion or stabilization of the bacterial community in the gastrointestinal tract of suckling piglets might especially be beneficial with regard to the critical weaning time, which should be investigated in future studies.

## Methods

### Study design

The experiment was approved according to the German Tierschutzgesetz by the Landesamt für Gesundheit und Soziales, Berlin, Germany. Primiparous sows (TOPIGS-SNW, Senden, Germany) were randomly divided into two groups. At the beginning of the study, the average age of the sows was 272 ± 24 days and the average body weight 146 ± 17.1 kg. The inulin group (n = 10) received a mash diet with the addition of 2.0% (gestation diet) or 2.2% (lactation diet) inulin (Prebiofeed 95, Speerstra Feed Ingredients BV, Lemmer, Netherlands). The inulin concentration in the non-supplemented diets was 1.0% (gestation diet) and 0.8% (lactation diet). The total concentration of 3.0% inulin in the diets was chosen based on literature evidence that this concentration can affect the host organism [21]. The control group (n = 11) received the same diets without the addition of inulin. The diets were offered from 21 days a.p. (gestation diet) until 14 days p.p. (lactation diet), with the change-over from the gestation diet to the lactation diet on day 1 p.p. Feed allowances were adjusted according to the maternal body weight and the number of piglets [22]. Water was offered *ad libitum*. The composition and nutrient characteristics of the experimental diets are described in Table 5. The sows were housed individually on straw beddings together with their litters. The suckling piglets only received colostrum and milk of their mothers, while no additional feed was offered. The treatment groups were kept in separate housing facilities under an identical light (12 hours light/12 hours darkness) and temperature (24°C) regimen.

**Table 5:** **Ingredients and analysed composition of the experimental diets**

		**Gestation diet**	**Lactation diet**
**Ingredients**			
Wheat	%	32.0	30.0
Barley	%	18.0	15.0
Peas	%	11.5	14.4
Beans, extruded	%	19.0	13.5
Soybeans	%	-	10.0
Wheat bran	%	13.0	8.40
Rapeseed cake	%	2.50	4.80
Premix	%	2.70	2.90
Sunflower oil	%	1.30	1.00
**Analysed composition**			
Crude protein	g/kg diet	161	185
Crude fat	g/kg diet	20.9	43.1
Crude fiber	g/kg diet	54.1	53.0
Nitrogen-free extract	g/kg diet	565	560
Crude ash	g/kg diet	46.8	49.6
Calcium	g/kg diet	7.45	8.59
Total phosphorus	g/kg diet	7.03	7.03
Sodium	g/kg diet	1.69	1.96
Magnesium	g/kg diet	1.28	1.96
Potassium	g/kg diet	6.80	8.58
Iron	mg/kg diet	277	298
Zinc	mg/kg diet	107	114
Copper	mg/kg diet	19.5	23.4
Inulin	g/kg diet	10.0^1^	8.00^1^

^1^Addition of inulin (Prebiofeed 95, Speerstra Feed Ingredients BV, Lemmer, Netherlands) for the inulin group: 20 g/kg diet (gestation diet) or 22 g/kg diet (lactation diet).

### Sampling procedure

The faeces of the sows were collected in the morning of day 4 a.p. and day 1 and day 5 p.p. The faeces were directly taken from the anus of the animals and stored at −80°C prior to further analysis.

The suckling piglets (n = 8/group) were euthanised on day 10 after birth. For anaesthesia, a combination of ketamine hydrochloride (Ursotamin®, Serumwerk Bernburg AG, Bernburg, Germany, 25 mg/kg body weight (BW); intramuscular injection) and azaperone (Stresnil®, Jansen-Cilag, Neuss, Germany, 2 mg/kg BW; intramuscular injection) was used. When the suckling piglets were narcotised, a combination of tetracaine hydrochloride, mebezonium iodide and embutramide (T61®, Intervet, Unterschleißheim, Germany, 0.5 ml/kg BW) was injected. Subsequently, the abdomen of the suckling piglets was opened to separate the gastrointestinal tract. Digesta samples of the stomach, small intestine, caecum and rectum were taken and stored at −80°C prior to further analysis.

### DNA-extraction and quantification of the bacteria in the digesta and faeces

The quantification of total eubacteria, enterobacteria, enterococci, bifidobacteria, lactobacilli, *L. reuteri, L. amylovorus, L. johnsonii, L. mucoase*, *C. leptum*, *C. coccoides* and the *Bacteroides-Prevotella-Porphyromonas* Cluster (BPP) in the faeces of the sows and digesta of the suckling piglets was accomplished by quantitative PCR (qPCR). Total nucleic acids were extracted by shearing 1 g sample with a 4 M guanidinisothiocyanate-solution and 3 g of glass beads in a bead beater. After a phenol-chloroform extraction, the nucleic acids were collected by isopropanol precipitation and purified with commercial spin columns (Macherey-Nagel, Düren, Germany). The DNA content was determined by fluorometric quantification (NanoDrop ND 3300, Fisher Scientific, Schwerte, Germany) with the Hoechst 33258 dye and calf thymus DNA as a reference. The cell numbers of eubacteria and enterobacteria were detected with a Taqman assay [23]. Lactobacilli [24], enterococci, bifidobacteria [25], *C. leptum, C. coccoides* [26] as well as *Lactobacillus reuteri*, *L. johnsonii* and *L. amylovorus* [27] were detected using the stated published primer sequences. Specific primers for *L. mucosae* (16S rRNA gene) were designed and validated at the Institute of Animal Nutrition, Berlin. All primers were purchased from MWG Biotech (Straubing, Germany). For the PCR amplification and fluorescent data collection, a Stratagene MX3000p (Stratagene, Amsterdam, The Netherlands) was used. The mastermix consisted of 12.5 μl Brilliant SYBR Green QPCR Mastermix (Stratagene, Amsterdam, The Netherlands) or 12.5 μl HotStartTaq Mastermix (Qiagen, Hilden, Germany) for Taqman-assays, 0.5 μl of each primer (10 μM), 0.75 μl ROX reference dye (1:500 diluted), and 10.75 μl water. One μl sample was added before PCR amplification. In order to activate the polymerase, all amplification programs included an initial denaturation step at 95°C for 15 min. All PCR programs featured an annealing time of 30 sec, and a 30 sec extension at 72°C. The annealing temperature for eubacterial cell numbers was 50°C. The detection of lactobacilli was carried out at 55°C annealing temperature. The quantification procedure is described in detail elsewhere [28]. In short, a series of autoclaved (1 h, 121°C, 2 bar) pig faeces samples was provided with different bacterial species and known cell numbers (10^9^ to 10^3^ cells/g wet weight). This quantification method employed extracts from a large number of reference strains inoculated in a sterile matrix and thus circumvents the bias of extraction efficiency and enables the expression of results as cell number per gram sample instead of target gene copy numbers. After extraction and purification, these extracts were used as PCR calibration samples and the results were expressed as cell number/g sample wet weight.

### pH, lactate, ammonia and short-chain fatty acids

The faecal and digesta samples were diluted with distilled water (1:10) and the pH of the samples was determined by using an electronic pH meter (Beckman Coulter, Inc, Fullerton, CA, USA).

For the measurement of D- and L-lactate, the samples were diluted with 1 M perchloric acid (1:5 w/v), centrifuged (1400 × g, 15 min) and stored at −20°C until enzymatic analyses using commercial kits (Boehringer, Mannheim, Germany).

The amounts of ammonia in the samples were analyzed colorimetrically using the Berthelot-reaction in microtitration plates. In brief, 20 μl of each sample were chlorinated with 100 μl of 0.2% alkaline hypochloride (Sigma Aldrich, Deisenhofen, Germany), resulting in the conversion of NH_3_ to chloramine (NH_2_Cl), in the following reaction with thymol to N-chlor-2-isopropyl-5-methyl chinon-monoimin and further to indophenol using 100 μl of 5% phenol nitroprusside (Sigma Aldrich). After reagent addition, the samples were incubated in the microtitration plates for 10 min and the extinction was measured at 620 nm in a Tecan Sunrise™ microplate reader (Tecan Austria GmbH, Grödig, Austria).

For the detection of the short-chain fatty acids, 300 mg of each sample was diluted with distilled water, homogenized, and centrifuged (Heraeus Instruments, Düsseldorf, Germany) at 11900 × g for 15 min. Hexanic acid was used as an internal standard (0.5 mmol/l). The sample (1.0 μl) was injected into a gas chromatograph (Model 19095 N-123, Agilent Technologies, CA, USA), fitted with a HP-INNOWax column A (length 30 m, internal diameter 530 μm with film thickness of 1.0 μm). The initial temperatures of the oven, injector and FID-detector were 70°C, 230°C and 250°C, respectively. Hydrogen gas, produced by a gas generator (Parker ChromGas, Parker Hannifin Corporation, MN, USA) was the carrier gas used at a flow rate of 30 ml/min.

### Statistical analysis

Data of the sows were analysed by two-factor analysis of variance (fixed factors diet, time and their interaction) using the GLM Repeated Measures procedure from SPSS 19 (SPSS Inc., Chicago, IL, USA) with time as within subject factor. Data of the suckling piglets were also analysed with SPSS 19. Normal distribution of the data was tested using Shapiro-Wilk-test, and data were compared in case of normal distribution with the t-test or in the case of not normally distributed data with the nonparametric Mann–Whitney-U-test. The data are presented in tables as mean and standard deviation. The significance level for group differences was *P ≤* 0.05.
